# Efficacy of unblinded and blinded intermittently scanned continuous glucose monitoring for glycemic control in adults with type 1 diabetes

**DOI:** 10.3389/fendo.2023.1110845

**Published:** 2023-02-22

**Authors:** Lixin Guo, Yuxiu Li, Mei Zhang, Xinhua Xiao, Hongyu Kuang, Tao Yang, Xiaofan Jia, Xianbo Zhang

**Affiliations:** ^1^ Department of Endocrinology, Beijing Hospital, National Center of Gerontology, Institute of Geriatric Medicine, Chinese Academy of Sciences, Beijing, China; ^2^ Department of Endocrinology, Peking Union Medical College Hospital, Peking Union Medical College, Chinese Academy of Medical Sciences, Beijing, China; ^3^ Department of Endocrinology and Metabolism, The First Affiliated Hospital with Nanjing Medical University, Nanjing, Jiangsu, China; ^4^ Department of Endocrinology, The First Affiliated Hospital of Harbin Medical University, Harbin, Heilongjiang, China

**Keywords:** clinical trial, continuous glucose monitor, sensors, type 1 diabetes, blinded and unblinded

## Abstract

**Objective:**

Intermittently scanned continuous glucose monitoring (isCGM) is used for unblinded or blinded monitoring of interstitial glucose. We aimed to compare the efficacy of blinded and unblinded isCGM with the FreeStyle Libre system for glycemic control in adults with type 1 diabetes (T1D).

**Research design and methods:**

This randomized clinical trial conducted between October 2018 and September 2019 across four endocrinology practices in China included 273 adults aged ≥18 years with T1D, who were randomly divided in a 2:1 ratio into the unblinded (n = 199) or blinded isCGM group (n = 78). In the blinded group, the clinician used FreeStyle Libre Pro system for monitoring, but self-monitoring was also performed by the patients.

**Results:**

Two hundred sixteen (78%) participants completed the study (152 [75%] in the unblinded and 64 [82%] in the blinded group). At 12 weeks, a significant increase in TIR (3.9-10.0 mmol/L) was only observed in the unblinded group, along with a significant decrease in hyperglycemia (>13.9 mmol/L), hypoglycemia (<3.0 mmol/L), glycemic variability. Further, the mean HbA1c reduction from baseline to 12 weeks was 0.5% in the unblinded isCGM group and 0.4% in the blinded isCGM group respectively (P < 0.001), but the significance did not remain after adjustment for between-group differences. Finally, 99.5% of the blinded isCGM values and 93.8% the of unblinded isCGM values were obtained at the final visit.

**Conclusions:**

The unblinded isCGM system was associated with benefits for glucose management, but nearly 100% of the attempted profiles were obtained successfully with the blinded isCGM system. Thus, combining real-time and retrospective data with isCGM might be the most impactful way to utilize flash glycemic monitoring devices.

## Introduction

Monitoring of glucose levels is essential for effective management of type 1 diabetes (T1D). Self-monitoring of blood glucose (SMBG) with glucose meters remains the mainstay of glycemic monitoring in T1D. However, this method can only provide point-in-time measurements of current glucose levels and does not indicate the trend in glucose levels. Therefore, silent glucose excursions could be missed with the SMBG method. In contrast, methods for continuous glucose monitoring (CGM) have been shown to have significant benefits in improving glycemic control in patients with type 1 and type 2 diabetes (1–3). In particular, they can help reduce the risk of hypoglycemia and hyperglycemia in patients with T1D (4–7).

CGM is an important adjunctive data collection strategy that provides a comprehensive 24-h glycemic profile compared to the relatively sparse information available with SMBG. Currently, three types of CGM devices are used in clinical practice: retrospective systems, real-time systems, and flash or intermittently viewed systems (8). Retrospective CGM systems are typically used in a blinded manner over a 3- 7 days wear period, and the data are reviewed retrospectively by clinicians. Real-time CGM devices are also used for short-term monitoring, but they are used in an unblinded manner. The data obtained enable patients and clinicians to respond to medication requirements in a timely way in order to prevent acute glycemic events, and the data are also useful in other areas of their daily diabetes self-management (9). Intermittently scanned CGM (isCGM) was developed for continuous monitoring of interstitial glucose and has a longer sensor life of 14 days, and it is often referred to as flash glucose monitoring.

FreeStyle Libre Flash Glucose (Abbott Diabetes Care, Alameda, CA) is the only isCGM system that is currently commercially available. The device is factory calibrated and does not need calibration against SMBG data over the course of the 14-day wear time. The use of isCGM has been associated with an increase in the amount of time in range (TIR), lower glycemic variability in randomized controlled trials with T1D cohorts, and reductions in hypoglycemia. Unlike real-time CGM systems that automatically transmit data to the patient’s receiver, isCGM requires the patient to swipe the receiver close to the sensor to obtain current and historical glucose data every 8 h (8). If there is a gap of more than 8 h between scans, only the data over the most recent 8 h will be retained and available for review. Overall, isCGM technology has made the collection, transmission, and monitoring of glucose data convenient.

The FreeStyle Libre Pro system for clinicians (blinded isCGM), which is available only in China, can automatically transmit data to the patient’s receiver; this method does not require the patient to scan the reading every 8 h and provides blinded retrospective data for up to 14 days (10). However, blinded CGM has not been convincingly proven to improve glycemic control (11, 12). Flash glycemic monitoring has been shown to improve glycemic control in adults with T1D, but no study so far has demonstrated the efficacy of blinded and unblinded isCGM in glycemic control. Person-reported outcomes (PROs) are usually assessed as secondary outcomes in glycemic technology studies. PROs show that the use of isCGM in adolescents can improve diabetes related distress with validated questionnaires. isCGM which allows greater benefits on psychological outcomes (13). However, several studies showed contradictory findings improvements associated with the use of glycemic technologies (14). In the current randomized study, for the first time, we have explored clinically meaningful data to determine the degree of agreement between the blinded and unblinded isCGM systems for T1D management in the real-world setting. Moreover, we used PRO to explore the benefits of technologies on psychological outcomes.

## Methods

### Study design and participants

**Figure 1:**
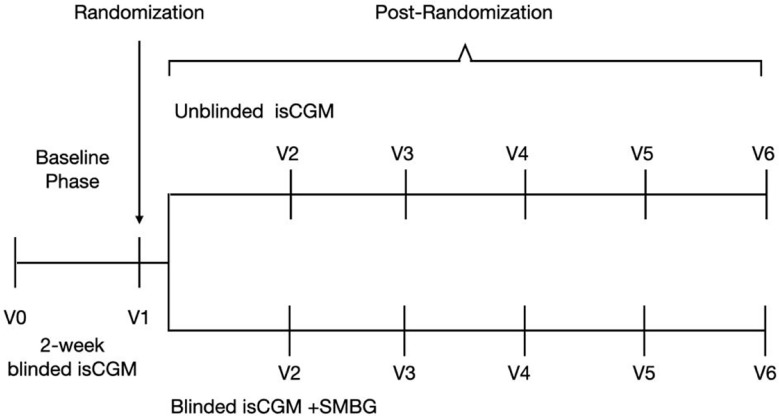
Study flow in a study of the efficacy of unblinded and blinded intermittently flash continuous glucose monitoring on glycemic control in adults with type 1 diabetes.

Adults with T1D were consecutively recruited for this 12-week, multi-center, prospective, 2:1 randomized controlled trial (**Figure 1**). The participants were recruited from four endocrinology practices in China, including Beijing Hospital, Peking Union Medical College Hospital, the First Affiliated Hospital of Nanjing Medicine University, and the First Affiliated Hospital of Harbin Medicine University.

The major eligibility criteria were clinical diagnosis of T1D, age ≥18 years, use of insulin therapy, and no use of CGM in the 3 months prior to enrollment. Willingness to participate in a 2-week screening period and use the blinded isCGM system were other inclusion criteria. In addition, the individual was required to perform SMBG at least four times a day (before every meal and before sleeping) with the blinded isCGM device.

The exclusion criteria were current or previous use of CGM or sensor-enhanced insulin pump therapy; known allergy to medical-grade adhesive; adverse events that endanger life or could cause death and serious systemic diseases; known severe diabetic retinopathy and/or macular edema; lactation, pregnancy, or intention to become pregnant during the study; presence of any condition that is likely to require MRI; use of medication containing acetaminophen or vitamin C; and unwillingness to use the study device.

During the study period, all the patients were free to use unblinded isCGM real-time glucose values or SMBG to adjust their diet, physical activity, and insulin therapy. All participating centers provided ethical approval for the study prior to its commencement, and all the participants provided their written informed consent.

### Procedures

This study was scheduled to include a total of six clinic visits—from the screening visit to the final visit (**Figure 1**). At the screening visit, the investigators obtained information about the medication history of the participants, preformed a physical examination, and completed patient-reported outcome assessments including AST, ALT, eGFR, urinary human chorionic gonadotropin, and electrocardiogram readings. A 2-h mixed meal tolerance test was performed, and blood samples were obtained for laboratory analysis of relevant parameters, including HbA1c, plasma glucose (glucose oxidase method, which was performed at each participating institute) and C-peptide (chemiluminescence analysis, which conducted at the central laboratory), at three time points (0 min, 60 min, and 120 min). Participants filled out the Chinese version of the scale for diabetes self-care activities (SDSCA), diabetes distress scale (DDS), hypoglycemia fear survey (HFS-II), and hypoglycemic confidence scale (HCS). At the second, fourth, and sixth visit, participants again underwent the physical examinations and completed the SDSCA, DDS, HFS-II, and HCS questionnaires. HbA1c was measured at the central laboratory in a randomized way and at 12 weeks, with the high-performance liquid chromatography method.

Over the 2-week measurement period, the eligible participants were randomly divided in a 2:1 ratio *via* a computer-generated sequence into the blinded isCGM group (clinician FreeStyle Libre Pro system), in which patients could use fingerstick blood glucose meter checks as needed, and the unblinded isCGM group (FreeStyle Libre system). The blinded isCGM group used the fingerstick blood glucose test data for management of glucose levels, while the unblinded isCGM group was required to scan the sensor at least three times a day. The participants, investigators, and staff were not blinded to the group allocation.

At each visit, participants in both study groups provided sensor glucose data, and the sensor was replaced. They also provided information about their daily diet, exercise, adverse events, and sensor insertion-site symptoms. Further, they received general diabetes management education and were provided with individualized treatment recommendations based on their glucose data (isCGM and SMBG data). Participants completed patient-reported outcome assessments prior to randomization and at 12 weeks.

### Outcomes

Outcomes were calculated at the follow-up visit based on data pooled over the 14-day measurement period after the screening visit and the 14 days prior to the final visit. The primary outcome was TIR or the percentage of time during which the glucose level was in the target range of ≥3.9-≤10 mmol/L from baseline to 12 weeks (15).

Secondary outcomes were changes in the percentage of time in which glucose level was in the range of >10.0- ≤13.9 mmol/L, > 13.9 mmol/L, in the range of ≥3.0-<3.9 mmol/L, and <3.0 mmol/L; coefficient of variation (CV); standard deviation (SD) and mean amplitude of glycemic excursions (MAGE); and HbA1c. The other secondary outcomes included patient-reported outcomes, namely, changes in daily dietary calories and proportions of carbohydrates and fat; changes in the number of daily steps; and changes in the SDSCA, DDS, HFS-II, and HCS scores.

The safety objective was to evaluate the safety of wearing the FreeStyle Libre Flash Glucose Monitoring System device in patients with T1D. Reportable adverse events included severe hypoglycemia (defined as an event that required assistance from another person due to altered consciousness), adverse events regardless of causality, and serious adverse events that require hospitalization, prolong hospitalization, cause disability, endanger life or result in death, or result in birth defects.

### Statistical analysis

A sample size of 216 participants was determined to detect a between-group difference in the target range (3.9–10 mmol/L), assuming a significant difference of an α-level of 0.05, power of 80% (β = 0.2), and a SD of 14. This number was increased to 270 participants to account for 20% with missing follow-up data.

All participants were analyzed according to their randomization group and included in the primary analysis. For the primary analysis, differences in the primary and secondary CGM outcomes between the final visit and screening visit in the two groups were assessed using paired *t*-tests. Missing data were managed with the direct likelihood method, which maximizes the likelihood function integrated over possible values of the missing data.

Analyses of prespecified secondary outcomes were conducted in parallel with the analysis of the primary outcome (CGM data were pooled across follow-up time points). Analysis of covariance was used to adjust for chance imbalances in baseline measurements between the treatment groups. Modification of the treatment effect by baseline variables was assessed by including an interaction term in the primary model. Secondary outcomes were analyzed by analysis of covariance of the differences between post-baseline and baseline values with study center, diabetes duration, baseline BMI, baseline SD, and baseline HbA1c as covariates in the two groups. Confidence intervals were calculated for the group least-square mean of each measure and the difference between group least-square means. Two-sided statistical tests were performed, and a significance of 0.05 was used in all tests.

The results were reported as the mean ± SD [minimum, maximum] or documented as the constituent ratio. Analyses were conducted with the SPSS 23.0 software.

## Results

### Clinical characteristics of the study participants

**Figure 2:**
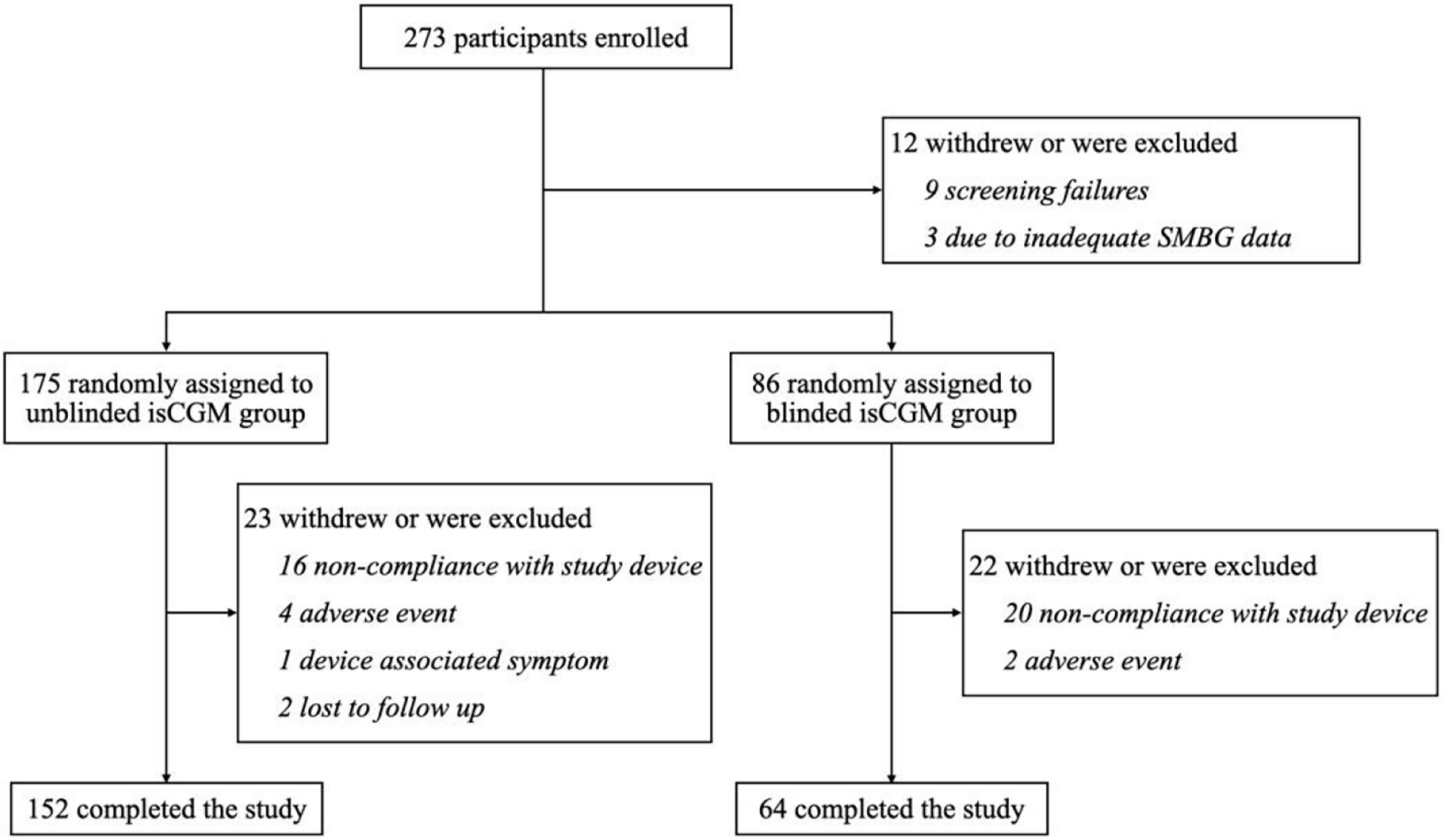
flow of participants in a study of the efficacy of unblinded and blinded intermittently flash continuous glucose monitoring on glycemic control in adults with type 1 diabetes.

From October 2018 to September 2019, a total of 273 eligible participants were randomly assigned to the unblinded isCGM (n = 199) group or the blinded isCGM group (n = 78). The 12-week visit was completed by 152 participants (75%) in the unblinded isCGM and 64 participants (82%) in the blinded isCGM group (**Figure 2**).

The included participants had comparable baseline characteristics (**Table 1**): There was no significant difference in age (mean = 40.8 years [range = 18–77] versus 42.6 years [range = 19–71]), duration of diabetes (mean = 10.0 years [range = 0–52.2] versus 10.2 years [range = 0.3–32.1]), proportion of females (58.8% versus 62.5%), use of multiple daily injections (80.3% versus 79.7%), HbA1c (mean ± SD = 8.0 ± 1.8% versus 7.7 ± 1.7%), and C-peptide levels (mean ± SD = 0.2 ± 0.4 ng/mL versus 0.2 ± 0.4 ng/mL) between the unblinded isCGM group and the blinded isCGM group (P > 0.05 for all the variables). No episodes of severe hypoglycemia or diabetic ketoacidosis were reported.

**Table 1:** Baseline characteristics of participants in a study of the efficacy of unblinded and blinded intermittently flash continuous glucose monitoring on glycemic control in adults with type 1 diabetes.

Characteristic	Unblinded isCGM(N=152)	Blinded isCGM (N=64)	P values
**Age,year, Mean(SD)[range]**	40.8 (14.4) [18-77]	42.6 (14.4) [19-71]	0.406
Diabetes duration, year			
**Mean(SD)[range]**	10.0 (9.5) [0.0-52.2]	10.2 (9.3) [0.28-32.14]	0.896
Sex			
***Female[n(%)]***	90 (58.8)	40 (62.5)	0.654
***Male[n(%)]***	63 (41.2)	24 (37.5)	/
**BMI, kg/m^2^, Mean(SD)[range]**	22.0 (2.5) [16.8-29.2]	21.3 (2.6) [16.7-32.7]	0.068
therapy			
***multiple daily injection[n(%)] ***	122 (80.3)	51 (79.7)	0.923
***Insulin pump use[n(%)]***	30 (19.6)	13 (20.3)	/
**HbA1c, %, Mean(SD)[range]**	8.0 (1.8) [5.0-15.2]	7.7 (1.7) [5.3-14.1]	0.256
**C-peptide, *Mean(SD)[range]***	0.2 (0.4) [0-2.7]	0.2 (0.4) [0-2.5]	0.980

### Comparison of scanning frequency and intra-day patterns

**Figure 3:**
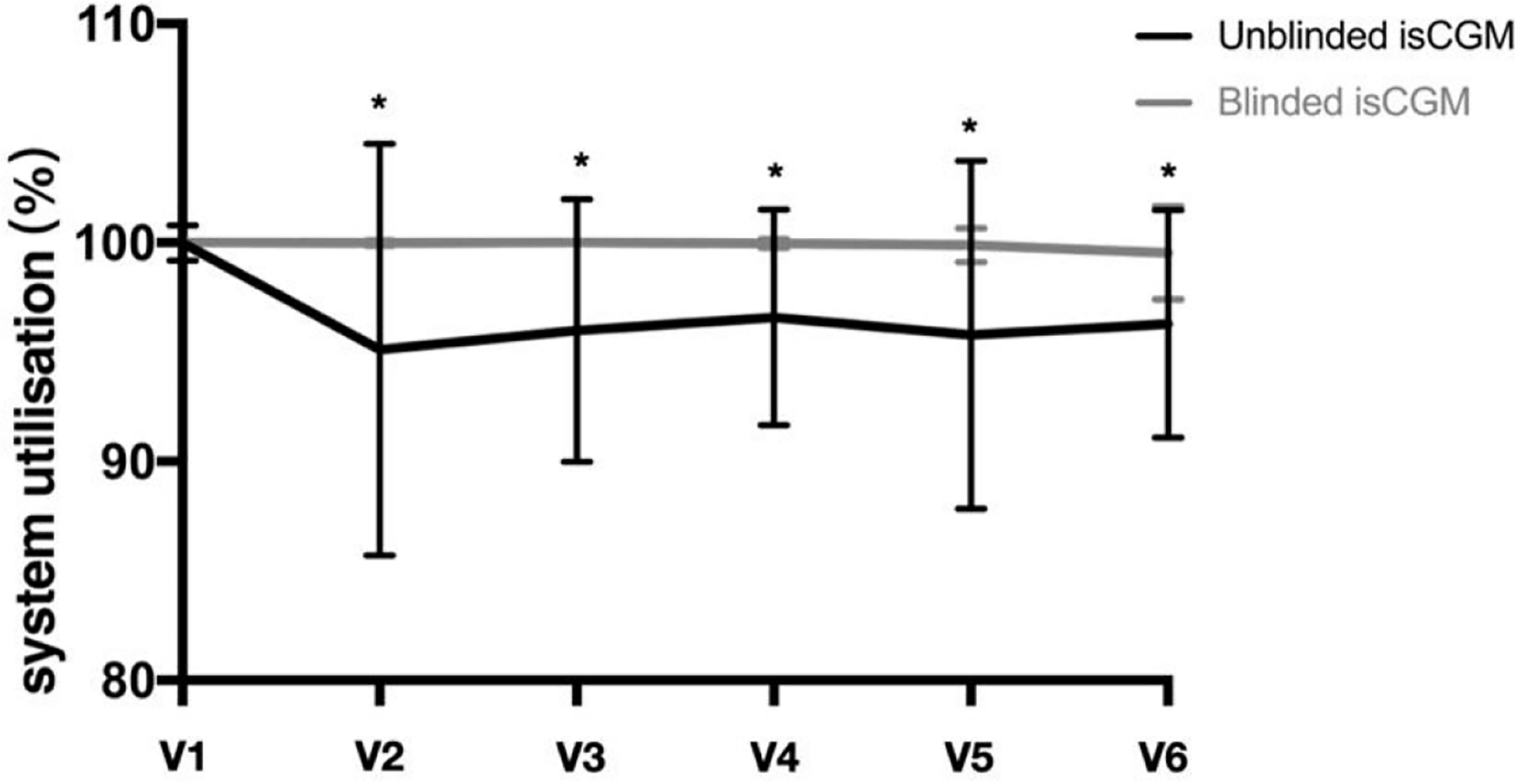
Glucose monitoring system utilization in a study of the efficacy of unblinded and blinded intermittently flash continuous glucose monitoring on glycemic control in adults with type 1 diabetes.*P<0.05 between unblinded isCGM and blinded isCGM.

**Figure 4:**
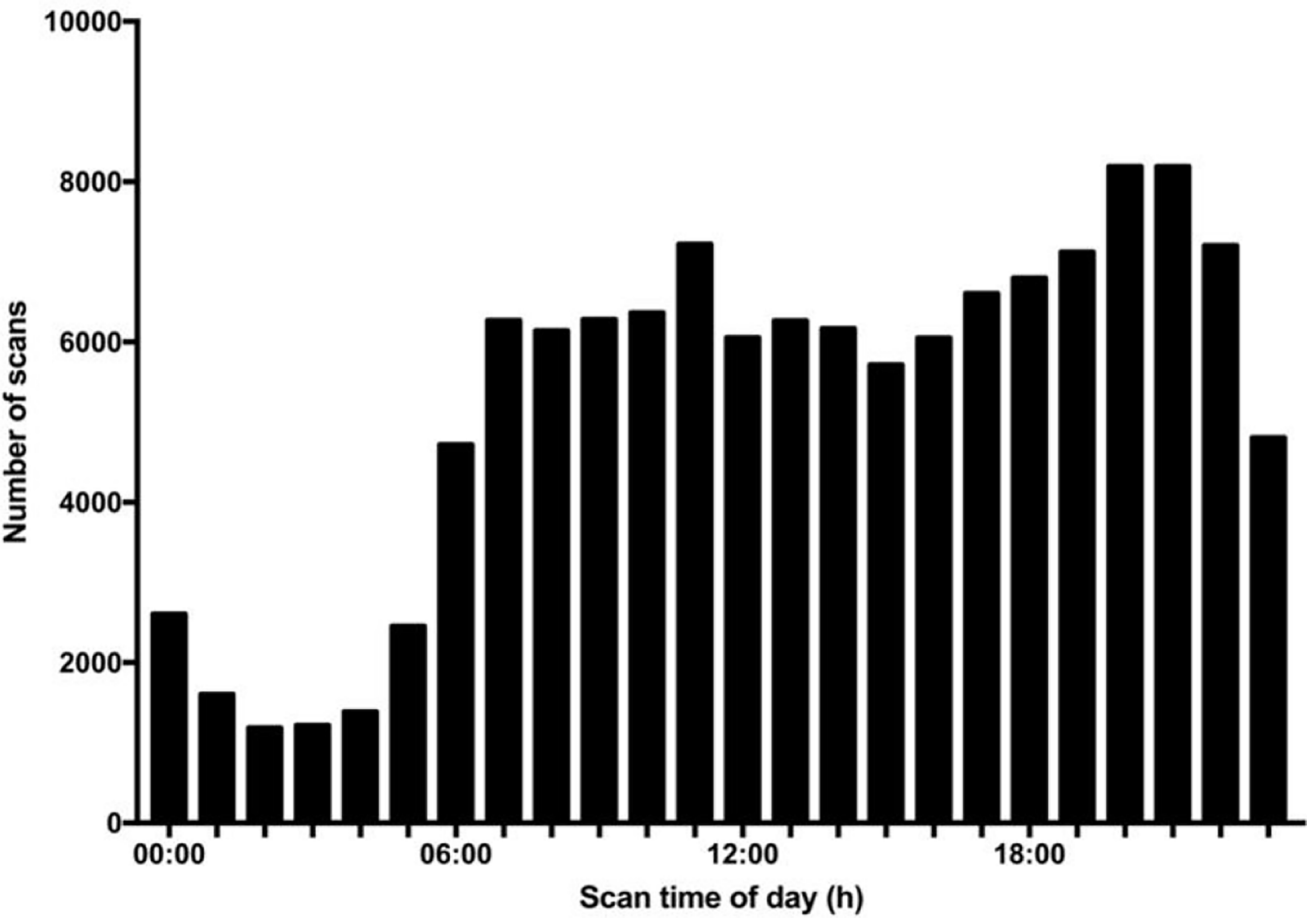
Glucose monitoring frequency in a study of the efficacy of unblinded and blinded intermittently flash continuous glucose monitoring on glycemic control in adults with type 1 diabetes. Total number of scans by time of day in the unblinded isCGM.

With regard to data reporting, 99.5% of the blinded isCGM values and 93.8% of the unblinded isCGM values were obtained at the final visit (**Figure 3**). Scanning was performed four times more often during typical awake hours (6 AM to 12 AM) than during typical sleeping periods (12 AM to 6 AM). Scanning was most frequently performed between 8 and 10 PM, while the frequency was the lowest at 2–3 AM. The pattern of daily scanning is shown in **Figure 4**.

### Glycemic metrics

**Figure 5:**
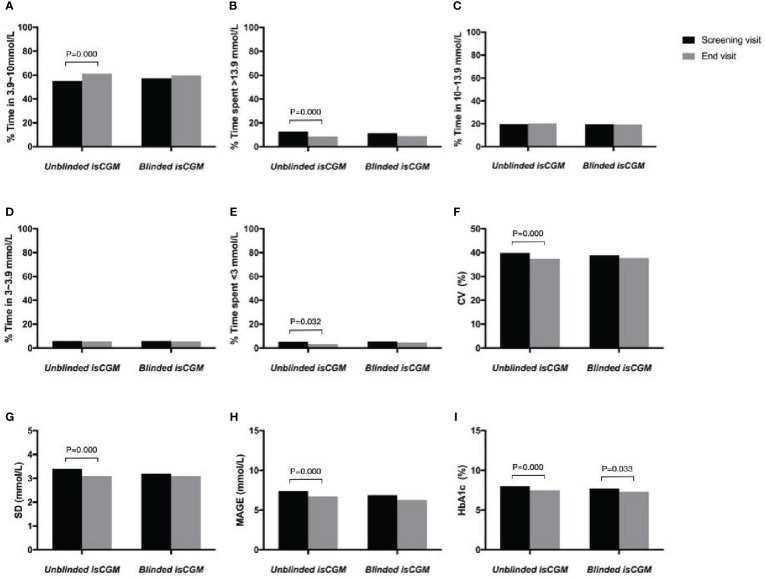
The efficacy of unblinded and blinded intermittently flash continuous glucose monitoring on glycemic control in adults with type 1 diabetes. Percentage of time in range **(A)**, hyperglycemia **(B, C)**, hypoglycemia **(D, E)**, Glucose variability **(F–H)** and HbA1c **(I)**.

The mean TIR percentage between 3.9 and 10 mmol/L was 55.2% at the baseline and 61.3% at 12 weeks in the unblinded isCGM group, and 57.4% at the baseline and 59.7% at 12 weeks in the blinded isCGM group. The values were significantly higher in the unblinded isCGM group (P < 0.001), but were not significant in the blinded isCGM group (**Table 2**, **Figure 5A**).

**Table 2:** Glycaemic and glucose variability outcomes in a study of the efficacy of unblinded and blinded intermittently flash continuous glucose monitoring on glycemic control in adults with type 1 diabetes.

	*Screening visit*		*End visit*		*difference in adjusted means in the unblinded and blinded group*	*P value*
	Unblinded isCGM (N=152)	Blinded isCGM (N=64)	Unblinded isCGM (N=152)	Blinded isCGM (N=64)		
*Sensor data *						
*** Percent of isCGM data***	99.2 (8.2)	99.9 (0.9)	93.8 (13.8)*	99.5 (2.1) †	-4.7 (-8.8, -0.6)	0.026
*Time in range *						
*** Glucose 3.9～10mmol/L (%)***	55.2 (20.28)	57.4 (19.56)	61.3 (18.08)*	59.7 (18.94)	3.3 (-2.2,8.82)	0.236
*Time in hyperglycemia*						
*** Glucose >13.9mmol/L (%)***	12.8 (17.42)	11.4 (16.69)	8.5 (11.01)*	9.1 (13.24)	-0.4 (-4.1,3.4)	0.849
*** Glucose >10～*** *≤* ***13.9mmol/L (%)***	19.8 (11.40)	19.6 (12.23)	20.4 (12.47)	19.3 (12.10)	1.2 (-2.4,4.7)	0.510
*Time in hypoglycemia*						
*** Glucose*** *≥* ***3.0～<3.9mmol/L (%)***	6.1 (4.90)	6.1 (5.72)	5.7 (5.02)	5.6 (4.40)	-0.1 (-1.5,1.4)	0.941
*** Glucose <3.0mmol/L (%)***	5.3 (10.79)	5.5 (7.80)	3.4 (4.59)*	4.7 (8.24)	-1.6 (-4.5,1.3)	0.280
*Glucose variability *						
*** CV (%)***	39.9 (8.4)	38.9 (9.4)	37.5 (7.4)*	37.8 (6.9)	-1.1 (-3.1,0.9)	0.299
*** SD (mmol/L)***	3.4 (1.11)	3.2 (1.11)	3.1 (0.95)*	3.1 (0.91)	-0.05 (-0.3,0.2)	0.692
*** MAGE (mmol/L)***	7.4 (2.30)	6.9 (2.33)	6.7 (2.25)*	6.3 (1.94)	0.1 (-0.5,0.6)	0.799
*** Number of hypoglycemia events***	15.5 (12.2)	11.0 (10.4)	13.2 (11.2)	10.8 (7.5)	-2.4 (-7.1,2.3)	0.318
*** HbA1c (%)***	8.00 (1.78)	7.7 (1.69)	7.5 (1.16)*	7.3 (1.30)*	-0.02 (-0.3,0.2)	0.861
*** difference in HbA1c compared to baseline<0.5% (n, %)***	–	–	126 (82.9)	50 (78.1)	–	0.445
*** difference in HbA1c compared to baseline<1.0% (n, %)***	–	–	144 (94.7)	62 (96.9)	–	0.727
*Psychological Quality Questionnaires*						
*** DDS***	34.5 (13.4)	33.6 (16.0)	31.5 (15.0)*	29.4 (15.0)*	1.1 (-3.2,5.4)	0.601
*** HFS-II***	10.4 (8.0)	8.2 (6.6)	10.0 (8.2)	10.0 (7.4)*	-2.1 (-4.2,-0.03)	0.046
*** HCS***	18.3 (6.0)	16.0 (7.0) †	16.8 (7.2)*	15.8 (7.4)	0.9 (-3.0,1.3)	0.431
*Self Management Questionnaires*						
*** SDSCA***	41.1 (12.4)	44.5 (16.4)	42.1 (16.8)	42.6 (17.6)	3.0 (-2.2,8.2)	0.255
*** Steps***	9933.0 (4198.4)	9614.3 (4147.9)	9143.5 (4200.1)*	8920.4 (4679.3)	-338.3 (-1803.4, 1126.8)	0.649
*Diet*						
*** Calorie (kcal)***	1363.3 (433.1)	1429.2 (418.1)	1396.1 (380.3)	1481.5 (886.5)	113.7 (-16.7,244.1)	0.087
*** Carbohydrates (%)***	51.0 (9.0)	51.8 (8.3)	49.6 (7.5)	49.9 (7.0)	0.6 (-3.1,4.3)	0.749
*** Protein (%)***	18.7 (5.3)	18.4 (3.0)	18.9 (3.5)	19.3 (4.1)	-0.5 (-2.3,1.1)	0.509
*** Fat (%)***	31.2 (8.4)	30.0 (7.1)	31.4 (6.7)	30.9 (6.1)	-0.04 (-3.1,3.1)	0.978

Values are mean ± SD.

*P<0.05 between screening visit and end visit in the unblinded isCGM group or blinded isCGM group.

†P<0.05 between unblinded isCGM group and blinded isCGM group in the screening visit or end visit.

The percentage of time in which hyperglycemia occurred (>13.9 mmol/L) was 12.8% at the baseline and 8.5% during follow-up in the blinded isCGM group, and 11.4% at the baseline and 9.1% at 12 weeks in the blinded isCGM group. The mean hyperglycemia time was significantly lower in the unblinded isCGM group (P < 0.001), but the difference between the baseline and 12-week values were not significantly different in the blinded isCGM group (**Table 2**, **Figure 5B**). The mean percentage of time in which the glucose levels were in the hypoglycemia range (10-13.9mmol/L) was not compare in the two groups (**Table 2**, **Figures 5C, D**). The mean percentage of time in the hypoglycemia range (<3.0 mmol/L) was 5.3% at the baseline and 3.4% at 12 weeks (P = 0.032) in the unblinded isCGM group, but the difference between the baseline and 12-week values were not significantly different in the blinded isCGM group (**Table 2**, **Figure 5E**).

The CV (-2.4%), SD (-0.3 mmol/L), and MAGE (-0.7 mmol/L) were significantly lower at 12 weeks in the unblinded isCGM group (P < 0.001), but these values did not decrease significantly compared to the baseline in the blinded isCGM group (**Figures 5F–H**).

Mean HbA1c was 8.0% at the baseline and 7.5% at 12 weeks in the unblinded isCGM group, and it was 7.7% at the baseline and 7.3% at 12 weeks in the blinded isCGM group. HbA1c showed a significant reduction of 0.5% in the unblinded isCGM group and 0.4% in the blinded isCGM group (P < 0.001 for both groups) (**Table 2**, **Figure 5I**).

After adjusting for between-group differences, no significant difference remained in the effect of the study treatment between the unblinded isCGM and blinded isCGM groups with regard to 12-week TIR, hypoglycemia time, hyperglycemia time, CV, SD, MAGE, and HbA1c (P > 0.05) (**Table 2**).

### Psychological questionnaires

The mean diabetes distress percentage was 34.5% at the baseline and 31.5% at 12 weeks in the unblinded isCGM group, and was 33.6% at the baseline and 29.4% at 12 weeks in the blinded isCGM group. Diabetes distress was significantly reduced from the baseline to 12 weeks in both groups (P < 0.05). Hypoglycemia fear behavior increased significantly from 8.2% at the baseline to 10.0% at 12 weeks in the blinded isCGM group (P < 0.05), but there was no significant change in the unblinded isCGM group. However, hypoglycemic confidence decreased from 18.3% at the baseline to 16.8% at 12 weeks in the unblinded isCGM group (P < 0.05). After adjusting for between-group differences, no significant difference remained between the unblinded isCGM and blinded isCGM groups (**Table 2**).

### Self-management questionnaires, steps, and diet

The questionnaire scores for SDSCA did not significantly favor either monitoring system. The number of daily steps was significantly reduced in the unblinded isCGM group (9933.0 ± 4198.4 *vs*. 9143.5 ± 4200.1, P < 0.05), while there was no significant difference in the blinded isCGM group (9614.3 ± 4147.9 *vs*. 8920.4 ± 4679.3, P > 0.05). There was no significant change in the self-management questionnaire scores for calories, carbohydrates, protein, and fat in either group (**Table 2**).

## Discussion

This prospective, randomized study was conducted to compare the unblinded and blinded isCGM glucose profiles in adults with T1D, and the findings showed that over 12 weeks of isCGM use is beneficial in the management of T1D.

Clinical application of CGM has been generally indicated to result in a significant improvement in diabetes management (8). However, some studies have shown that retrospective CGM systems do not improve glycemic control. A study on 102 patients with T1D in a 3-day blinded CGM trial with iPRO (Medtronic, Northridge, CA) did not find any significant improvement in HbA1c for up to 7 months after the CGM device was worn (16). Another study did not find a significant difference in HbA1c levels in patients with T1D when those using SMBG were compared with those using a 72-h blinded CGM device (11). However, retrospective CGM systems have been found to be valuable for collecting detailed glycemic excursion data (17).

Real-time CGM devices enable patients to respond immediately to mitigate or prevent acute glycemic events and allow patients to make better informed decisions about their medication requirements and other areas of their daily diabetes self-management. In the IMPACT study, the use of an intermittently viewed system was associated with a reduction in hypoglycemia as compared with a conventional SMBG device in adults with well-controlled T1D (1, 3). This indicates that increasing the frequency of glucose monitoring is sufficient to reduce hypoglycemic risk, even in the absence of alarms. The isCGM system provides actual and unblinded interstitial glucose concentrations, but the earlier generation of isCGM devices required patients to perform a sensor scan every 8 h. If more than 8 h elapsed between scans, the device would only display a plot profile of the last 8 h. Missing data in the isCGM system cannot be recovered after the fact. Therefore, one of the challenges with this system is to determine whether CGM data collection has been successful in real time versus after the CGM process has been completed.

Unlike unblinded isCGM, blinded isCGM can automatically transmit data to the patient’s receiver and provides blinded retrospective data for up to 14 days (10, 18). A key strength of our study was the use of the clinician isCGM systems. So far, no study has reported the efficacy of blinded and unblinded isCGM for glycemic control.

According to recent international consensus, individuals with T1D should strive to achieve 4% of time below the target range (<3.9 mmol/L), >70% of time within the target range (3.9–10.0 mmol/L), and <25% above the range (>10.0 mmol/L), with a glycemic variability (%CV) of <36% (18, 19). In our study, compared with the baseline phase, unblinded isCGM use was associated with a significantly greater TIR percentage, which increased from 55.2% at the baseline to 61.3% at the end of the study. We also found lower values for hyperglycemia time (>13.9 mmol/L), hypoglycemia time (<3.0 mmol/L), CV, SD, and MAGE in the unblinded isCGM users. Further, both isCGM systems resulted in a significant reduction in HbA1c. Taken together, these data indicate that while both blood glucose monitoring methods could improve blood glucose control, unblinded isCGM could increase the TIR while reducing time above range, time below range, and glycemic variability. Thus, the use of these variables for generating predictive alerts might result in even greater glycemic improvements. However, after adjustment for between-group differences, no significant difference was found in the effect of study treatment at 12 weeks between the unblinded isCGM and blinded isCGM groups in terms of 12-week HbA1c, TIR, hypoglycemia time, hyperglycemic time, CV, SD, MAGE, and HbA1c (P > 0.05). This emphasizes the greater challenges that are present in the management of T1D in the real world.

In the present study, we found that that 99.5% of the blinded isCGM values were obtained, compared with only 93.8% of the unblinded isCGM values at the final visit. The data showed that the majority of scanning was conducted during the awake hours spanning 6–18 h, while only a few scans were performed over the night-time hours spanning 0–6 h. The possible reasons for missing data in the unblinded isCGM group may be scanning frequency and the time of day for measurements according to the patient’s age, lifestyle, eating habits, level of physical activity, and understanding and motivation with regard to maintenance of glucose monitoring (20). The use of safety features may contribute to avoiding missing abnormal glycemic data and further improving glycemic control.

Diet, physical exercise, and psychological reactions are important components in the management of T1D across a patient’s lifespan (21, 22). Therefore, in this study, we also explored data on these self-managed key aspects. During the study period, both groups of patients were free to use unblinded isCGM real-time glucose values and SMBG to adjust their diet, physical activity, and insulin therapy. The results showed that the number of daily steps was reduced in the unblinded isCGM group, while there was no difference in the blinded isCGM group at the end of the study. However, there was no change in calorie, carbohydrate, protein, and fat consumption in both groups. The challenging management of diabetes could result in diabetes distress and risk for psychological disorders. However, the real-world study showed no significant association of CGM use and the level of diabetes distress (23). Our study showed that the participants of both groups reported improved diabetics distress, especially unblinded isCGM users. Our findings suggest that technology use, at least in the short term, may reduce diabetes distress. However, our findings also indicated that technology couldn’t address every aspect of living with diabetes. Not only individuals with T1D but also healthcare professionals should be involved in the interpretation of data in order to maximize the technological potential of these devices and improve their efficiency.

A major limitation of our study is that the intervention period of 12 weeks is relatively short. An extended monitoring period may provide insight into longer-term use of CGM and reflect the real-world setting. Additionally, these results also need to be confirmed in a large study population.

In conclusion, the use of isCGM systems resulted in a decrease in HbA1c level over 12 weeks among the adults with T1D in this study. The unblinded isCGM system was associated with benefits for glucose management, but with the blinded system, nearly 100% of the profiles were obtained successfully. It appears that the blinded isCGM systems can overcome both expected and unexpected data collection hurdles. Thus, combining both real-time and retrospective data gathered by isCGM might be the most appropriate and impactful way to utilize flash glycemic monitoring devices. However, further research is needed to understand the clinical importance of this finding and the applicability of these systems in the real world.

## Data availability statement

The original contributions presented in the study are included in the article/Supplementary Material. Further inquiries can be directed to the corresponding author.

## Ethics statement

The studies involving human participants were reviewed and approved by the ethics committees of Beijing Hospital, Peking Union Medical College Hospital, the First Affiliated Hospital of Nanjing Medicine University, and the First Affiliated Hospital of Harbin Medicine University. The patients/participants provided their written informed consent to participate in this study.

## Author contributions

LG, YL, XX, HK, and TY directed the study and were responsible for study design. LG, YL, MZ, XX, HK, TY, XJ, and XZ contributed to the project recruitment. MZ performed statistical analyses and drafted the initial manuscript. LG, YL, MZ, XX, HK, and TY contributed to the discussion and helped edit the manuscript and suggested revisions. LG, YL, MZ, XX, HK, TY, XJ, and XZ approved the final manuscript. LG is the guarantor of this work and, as such, had full access to all the data in the study and takes responsibility for the integrity of the data and the accuracy of the data analysis. All authors contributed to the article and approved the submitted version.
